# ACE Gene I/D Polymorphism and Obesity in 1,574 Patients with Type 2 Diabetes Mellitus

**DOI:** 10.1155/2016/7420540

**Published:** 2016-12-26

**Authors:** Yan-Hong Pan, Min Wang, Yan-Mei Huang, Ying-Hui Wang, Yin-Ling Chen, Li-Jun Geng, Xiao-Xi Zhang, Hai-Lu Zhao

**Affiliations:** ^1^Center for Diabetic Systems Medicine, Guangxi Key Laboratory of Excellence, Guilin Medical University, Guilin 541004, China; ^2^Institute of Basic Medical Sciences, Guilin Medical University, Guilin 541004, China; ^3^Department of Immunology, Faculty of Basic Medicine, Guilin Medical University, Guilin 541004, China

## Abstract

Association between ACE gene I/D polymorphism and the risk of overweight/obesity remains controversial. We investigated the possible relationship between ACE gene I/D polymorphism and obesity in Chinese type 2 diabetes mellitus (T2DM) patients. In this study, obesity was defined as a body mass index (BMI) value ≥ 25 kg/m^2^ and subjects were classified into 4 groups (lean, normal, overweight, and obese). PCR (polymerase chain reaction) was used to detect the ACE gene I/D polymorphism in T2DM patients. Metabolic measurements including blood glucose, lipid profile, and blood pressure were obtained. Frequencies of the ACE genotypes (DD, ID, and II) were not significant among the 4 groups of BMI-defined patients (*P* = 0.679) while ACE II carriers showed higher systolic blood pressure (SBP) and pulse pressure (PP) (all *P* < 0.050). Hyperglycemia, hypertension, and dyslipidemia in these T2DM patients were found to be significantly associated with BMI. In conclusion, the relationship of ACE gene I/D polymorphism with obesity is insignificant in Chinese patients with T2DM. SBP and PP might be higher in the ACE II carriers than in the DD and ID carriers.

## 1. Introduction

Angiotensin converting enzyme (ACE), a key enzyme in the renin angiotensin system (RAS), can convert angiotensin I (Ang I) into vasoconstrictor molecule angiotensin II (Ang II) [1]. Body fat and body weight could be raised and lowered accordingly by stimulating and inhibiting the production of Ang II, suggesting a possible link between ACE and obesity [2]. In 1990, Rigat B firstly described the polymorphism of ACE gene characterized by the presence (I) or absence (D) of a 287 bp Alu repeat sequence within intron 16 [3]. It was pointed out that ACE gene I/D polymorphism is involved in blood pressure (BP) and body fatness [4–6]. Thereafter, numerous studies focused on the association between ACE gene I/D polymorphism and hypertension [7, 8]. However, large samples of epidemiological studies that focus on the relationship of ACE gene I/D polymorphism and obesity are lacking. Body mass index (BMI) is recommended as a crucial indicator for obesity definition based on the WHO guidelines [9]. Thus, we have conducted a cross-sectional study to identify the association between obesity and ACE gene I/D polymorphism in Chinese patients with type 2 diabetes mellitus (T2DM).

## 2. Methods

### 2.1. Subjects and Ethics

All the subjects were newly diagnosed T2DM patients between January 2012 and June 2015 from the teaching hospitals of Guilin Medical University. A study's power calculation using the ratio of obesity and hypertension was done separately at the beginning of our work. By applying the sample estimate formula, we found that a sample size between 90 and 140 indicates a proper evaluation of the validity of the findings; thus, our participants (1,574 patients) were validated. And none of the patients recruited in the study were under antidiabetes and antihypertensive medications. The patients signed informed consent and an ethical approval was obtained from the institutional clinical research review committee. The diagnosis of T2DM was based upon the American Diabetes Association (ADA) criteria [10].

### 2.2. Metabolic Measurements

The metabolic variables included the definition of obesity (BMI and waist-to-hip ratio (WHR)), blood glucose (fasting plasma glucose (FPG) and glycated hemoglobin (HbA_1c_)), lipid profile (total cholesterol (TC), triglycerides (TG), low-density lipoprotein cholesterol (LDL-C), and high-density lipoprotein cholesterol (HDL-C)), and blood pressure (systolic blood pressure (SBP), diastolic blood pressure (DBP), mean arterial pressure (MAP), and pulse pressure (PP)).

### 2.3. Data Collection and Laboratory Tests

Clinical data including age, sex, body weight, height, waist and hip circumference, and BP were collected for analysis. BMI was calculated according to the following formula: BMI = body weight (in kilograms)/square of the height (in meters). Patients were divided into four groups following the standard of adults' BMI of Asian populations [11, 12]. WHR was calculated with waist circumference and hip circumference: WHR = waist/hip circumference (cm). Clinical data (FPG, HbA_1c_, TC, TG, LDL-C, and HDL-C) were obtained by laboratory tests. Clinical and biochemical data were acquired as described previously [13]: an automated ion-exchange chromatographic method was used to measure HbA_1c_ (reference range: 5.1–6.4%; Bio-Rad, Hercules, CA). Coefficient of variation (CV) between interassay and intra-assay cases for HbA_1c_ was ≤ 3.1% at values < 6.5%. Enzymatic methods were used to detect TC, TG, and HDL-C measurements on the Hitachi 911 automated analyzer. Friedewald's formula for triglyceride levels < 4.5 mmol/L was used to calculate LDL-C [14]. Both proper procedures and recommendations were used to measure resting BP as standard. We used the means of three sitting blood pressure readings taken one minute apart after 5 to 10 minutes of rest, using a digital blood pressure monitor [15]. Hypertension was defined according to a report of the expert committee on the diagnosis and classification of T2DM [16], SBP ≥ 140 mmHg and/or DBP ≥ 90 mmHg. MAP = (SBP + 2 × DBP)/3 (mmHg), and PP = SBP − DBP (mmHg).

### 2.4. Genetic Analysis

Genotyping for the ACE gene I/D polymorphism was performed using a PCR method as previously described [17]. The presence of II genotype produces a 490 bp fragment while in DD genotype a 190 bp fragment was confirmed, and the presence of both 490 and 190 bp fragments implied ID genotype.

### 2.5. Statistical Analysis

All data were expressed as means ± standard deviation [SD], frequency, or percentage, as appropriate. Hardy-Weinberg equilibrium was calculated using the gene-counting method and difference was assessed by Chi square (*χ*^2^) test. For continuous variables in normal distribution, one-way ANOVA was used to evaluate the difference among groups. All data were assessed using the PASW Statistics software 18.0 (SPSS Inc., Chicago, IL, USA). A two-tailed *P* < 0.05 was considered to be statistically significant.

## 3. Results

A total of 1,574 patients were enrolled in this research. Of these newly diagnosed patients, 40.53% were male, 35.13% were hypertensive, and 27.06% were obese. Frequencies of ACE gene I/D polymorphism were in Hardy-Weinberg equilibrium, indicating a homogeneous and representative sample population. Frequencies of I and D alleles were 67.25% and 32.75%, respectively. The genotype frequencies were 11.18% for ID, 43.14% for II, and 45.68% for DD accordingly.

### 3.1. The Distribution of ACE Gene Polymorphism according to BMI Multiclassification in T2DM Population

Table 1 shows genotypes and alleles distribution of ACE gene I/D polymorphism in T2DM patients according to BMI categories. Association between the ACE polymorphism and the BMI-defined overweight and obesity was insignificant based on our analysis.

### 3.2. The Distribution of Hypertension according to BMI Multiclassification in T2DM Population

An association between hypertension and BMI was identified in our study (Table 2). The patients with higher BMI occupied higher proportion of hypertension (15.52, 35.66, 40.86, and 41.25% for lean, normal, overweight, and obese, resp.).

### 3.3. The Distribution of Hypertension and ACE Gene Polymorphism in T2DM Population

The relationships between ACE genotypes and hypertension and between ACE gene alleles and hypertension were demonstrated in Tables 3 and 4 and negative outcomes were found. The proportion of I allele in hypertension was higher than the D allele though it has no statistical significance.

### 3.4. Clinical and Biochemical Characteristics of BMI Multiclassification and ACE Gene I/D Polymorphism

Tables 5 and 6 summarize the clinical and biochemical profiles of patients according to BMI categories and ACE genotypes. Compared with the normal BMI group, obesity and overweight patients tended to have higher WHR, FPG, HbA_1c_, TC, TG, LDL-C, SBP, DBP, and MAP values, but lower HDL-C level. Interestingly, ACE gene II carriers showed higher SBP and PP compared to DD and ID carriers in these Chinese T2DM subjects.

## 4. Discussion

To the best of our knowledge, this is the first large-scale epidemiological study to explore the relationship of ACE gene I/D polymorphism with obesity in Chinese patients with T2DM. Additionally, associations between the ACE gene I/D polymorphism and hypertension were analyzed. We found that there was no statistical significance in the relationship of ACE gene I/D polymorphism with BMI-based obesity, whereas ACE II carriers showed higher SBP and PP than those of DD and ID carriers.

It is increasingly recognized that obesity has been identified as a key risk factor for hypertension [18], dyslipidemia [19], and T2DM [20, 21]. However, it is still unclear whether there are complex interactions between genetic predisposition and environmental factors in the development of obesity. BMI has been considered as the key indicator in the identification of overweight and obesity, and increasing evidence of body fat composition, especially abdominal adipose tissue and ectopic fat deposition, was set [5, 22]. So, we explored the relationship between ACE gene I/D polymorphism and obesity based on BMI multiclassification. According to our findings, the relationship of ACE gene I/D polymorphism with BMI-defined obesity is insignificant in Chinese patients with T2DM; the obese group obtained higher ratio of hypertension. Furthermore, we also strengthened the fact that BMI is associated with hyperglycemia, hypertension, and dyslipidemia in T2DM patients [23, 24].

In addition, our results indicated that the ACE II carriers had higher SBP and PP than those of DD and ID carriers; this was a novel finding which was not reported in adults. In accordance with our results, one prior research reported that children carrying the II genotype were demonstrated to have higher BP and greater early growth acceleration compared to those with the other ACE genotypes [25]. Conversely, another study identified significantly higher SBP and MAP among those overfat D allele carrier subjects, compared to the normal fat D allele carriers and normal fat II subjects [26]. Besides, they also pointed out DD carriers with heavier BMI than those II individuals [26]. Other perspectives hold that ACE genotypes were not linked to BMI and obesity [27–29]. The controversies may be attributed to clinical characteristics of enrolled subjects, such as patients' age, racial difference, and sample size. Our large sample sizes gained a more credible result undoubtedly in Asians. Based on our results, there is a trend in favor of the insignificant relationship between BMI and ACE gene I/D polymorphism, and this equivocal outcome may account for the different BMI cut-off points adopted by researchers to define overweight and obesity. The polled results implied that the physiological environment and internal metabolism played a key role in the development of obesity and hypertension in T2DM [21].

*Study Limitations*. One limitation in our research was that we mainly focused on a polymorphism point. It is well known that obesity and T2DM are polygenic disorders and have a multifactorial influence, as evidenced by the various disease outcomes modulated by the gene-gene and gene-environment interactions, and thus a combination of other gene polymorphisms was considerable. It is also worth pointing out that our analysis was a transversal and monocenter research in the study design. Further longitudinal studies need to be performed to strengthen our result.

In conclusion, SBP and PP might be higher in the ACE II carriers than those in DD and ID carriers. The relationship of ACE gene I/D polymorphism with obesity was insignificant in Chinese patients with T2DM.

## Figures and Tables

**Table 1:** ACE gene I/D polymorphism relating to BMI in 1,574 Chinese patients with type 2 diabetes.

	Lean	Normal	Overweight	Obese	*P*
DD	5 (8.6)	76 (12.0)	34 (12.2)	61 (10.1)	0.679
ID	27 (46.6)	273 (43.3)	127 (45.5)	252 (41.6)	
II	26 (44.8)	282 (44.7)	118 (42.3)	293 (48.3)	
Total	58 (100)	631 (100)	279 (100)	606 (100)	
D	37 (31.9)	425 (33.7)	195 (35.0)	374 (30.9)	0.293
I	79 (68.1)	837 (66.3)	363 (65.0)	838 (69.1)	
Total	116 (100)	1262 (100)	558 (100)	1212 (100)	

Data are shown as number (percentage). *P* value is derived by the Chi square (*χ*^2^) test.

BMI: body mass index, defined as the weight in kilograms divided by the square of the height in meters. BMI is classified into 4 groups: lean (BMI < 18.5 kg/m^2^), normal (18.5 kg/m^2^≤ BMI < 23 kg/m^2^), overweight (23 kg/m^2^ ≤ BMI < 25 kg/m^2^), and obese (BMI ≥ 25 kg/m^2^).

**Table 2:** The association of hypertension and BMI.

	Normotensive	Hypertensive	*P*
Lean (BMI < 18.5)	49 (84.5)	9 (15.5)	0.001
Normal (18.5 ≤ BMI < 23)	406 (64.3)	225 (35.7)	
Overweight (23 ≤ BMI < 25)	165 (59.1)	114 (40.9)	
Obese (BMI ≥ 25)	356 (58.8)	250 (41.2)	

Data are shown as number (percentage). *P* value is derived by the Chi square (*χ*^2^) test.

BMI: body mass index, defined as the weight in kilograms divided by the square of the height in meters. BMI is classified to 4 groups: lean (BMI < 18.5 kg/m^2^), normal (18.5 kg/m^2^ ≤ BMI < 23 kg/m^2^), overweight (23 kg/m^2^ ≤ BMI < 25 kg/m^2^), and obese (BMI ≥ 25 kg/m^2^).

**Table 3:** The distribution of ACE genotypes relating to hypertension.

	DD	ID	II	*P*
Normotensive	114 (64.8)	431 (63.5)	431 (59.9)	0.181
Hypertensive	62 (35.2)	248 (36.5)	288 (40.1)	
Total	176 (100)	679 (100)	719 (100)	

Data are shown as number (percentage). *P* value is derived by the Chi square (*χ*^2^) test.

The definition of hypertension is systolic blood pressure (SBP) ≥ 140 mmHg and/or diastolic blood pressure (DBP) ≥ 90 mmHg.

**Table 4:** The distribution of ACE gene alleles relating to hypertension.

	Normotensive	Hypertensive	*P*
D	659 (33.8)	372 (31.1)	0.123
I	1293 (66.2)	824 (68.9)	
Total	1952 (100)	1196 (100)	

Data are shown as number (percentage). *P* value is derived by the Chi square (*χ*^2^) test.

The definition of hypertensive is systolic blood pressure (SBP) ≥ 140 mmHg and/or diastolic blood pressure (DBP) ≥ 90 mmHg.

**Table 5:** Clinical and biochemical characteristics of Chinese patients with type 2 diabetes relating to BMI.

	Lean	Normal	Overweight	Obese	*P* ^a^	*P* ^b^	*P* ^c^
Patients (*n*)	58	631	279	606	**—**	**—**	**—**
M : F	22 : 36	185 : 276	165 : 207	266 : 417	**—**	**—**	**—**
Age (years)	50.40 ± 14.91	54.80 ± 13.14	55.04 ± 13.28	52.73 ± 14.01	**0.021**	0.797	0.012
BMI (kg/m^2^)	17.23 ± 1.26	21.47 ± 1.11	24.05 ± 0.58	28.10 ± 2.81	**<0.001**	**<0.001**	**<0.001**
WHR	0.80 ± 0.07	0.86 ± 0.08	0.88 ± 0.06	0.90 ± 0.07	**<0.001**	**<0.001**	**<0.001**
FPG (mmol/L)	8.64 ± 3.25	8.78 ± 3.63	8.91 ± 3.50	9.78 ± 5.10	0.074	0.594	0.198
HbA_1c_ (%)	7.77 ± 1.70	7.80 ± 1.91	7.98 ± 2.18	8.44 ± 3.10	0.093	0.192	0.086
TC (mmol/L)	5.56 ± 1.60	5.32 ± 1.15	5.55 ± 1.28	5.60 ± 1.30	0.179	**0.001**	**0.003**
TG (mmol/L)	1.17 ± 0.65	1.38 ± 1.06	1.78 ± 1.39	1.90 ± 1.39	0.224	**<0.001**	**<0.001**
LDL-C (mmol/L)	3.38 ± 1.19	3.35 ± 0.97	3.49 ± 1.03	3.50 ± 1.03	0.828	**0.035**	**0.025**
HDL-C (mmol/L)	1.53 ± 0.42	1.33 ± 0.38	1.25 ± 0.35	1.19 ± 0.34	**<0.001**	**0.002**	**<0.001**
SBP (mmHg)	121.67 ± 20.27	132.20 ± 22.56	134.99 ± 21.75	136.10 ± 22.68	**0.001**	**0.012**	**0.037**
DBP (mmHg)	74.38 ± 8.46	78.18 ± 10.87	80.67 ± 11.18	82.08 ± 11.68	**0.015**	**0.001**	**<0.001**
MAP (mmHg)	90.14 ± 11.31	96.19 ± 13.46	99.15 ± 13.79	99.72 ± 13.94	**0.002**	**0.002**	**<0.001**
PP (mmHg)	47.29 ± 15.98	54.02 ± 17.4	55.43 ± 17.05	52.9 ± 15.67	**0.004**	0.223	0.260

Data are shown as means ± SD. *P* value is derived by analysis of variance (ANOVA).

^a^ *P* values compared normal group with lean group.

^b^ *P* values compared normal group with overweight group.

^c^ *P* values compared normal group with obese group.

BMI: body mass index; WHR: waist-to-hip ratio; FPG: fasting plasma glucose; HbA_1c_: glycated hemoglobin; TC: total cholesterol; TG: triglycerides; LDL-C: low-density lipoprotein cholesterol; HDL-C: high-density lipoprotein cholesterol; SBP: systolic blood pressure; DBP: diastolic blood pressure; MAP: mean arterial pressure; PP: pulse pressure.

**Table 6:** Clinical and biochemical characteristics of 1,574 Chinese patients with type 2 diabetes relating to ACE I/D polymorphism.

	DD	ID	II	*P* ^a^	*P* ^b^	*P* ^c^
Patients (*n*)	176	679	719	**—**	**—**	**—**
M : F	74 : 102	261 : 418	303 : 416	**—**	**—**	**—**
Age (years)	54.09 ± 13.90	53.27 ± 14.03	54.22 ± 13.26	0.912	0.195	0.26
BMI (kg/m^2^)	24.76 ± 3.41	24.86 ± 3.88	24.76 ± 3.73	0.982	0.611	0.663
WHR	0.88 ± 0.06	0.88 ± 0.09	0.88 ± 0.07	0.529	0.837	0.965
FPG (mmol/L)	9.08 ± 3.61	8.62 ± 3.37	8.88 ± 3.59	0.494	0.165	0.352
HbA_1c_ (%)	8.01 ± 2.13	7.80 ± 1.91	7.89 ± 1.98	0.452	0.406	0.66
TC (mmol/L)	5.48 ± 1.35	5.51 ± 1.25	5.48 ± 1.26	0.947	0.710	0.772
TG (mmol/L)	1.70 ± 1.33	1.65 ± 1.25	1.73 ± 1.34	0.736	0.222	0.254
LDL-C (mmol/L)	3.44 ± 0.97	3.49 ± 1.08	3.41 ± 0.97	0.716	0.167	0.198
HDL-C (mmol/L)	1.25 ± 4	1.28 ± 0.38	1.25 ± 0.36	0.825	0.132	0.18
SBP (mmHg)	132.45 ± 20.50	133.00 ± 23.11	135.20 ± 21.95	0.144	0.066	**0.041**
DBP (mmHg)	80.94 ± 11.18	79.76 ± 11.62	80.70 ± 11.22	0.801	0.123	0.226
MAP (mmHg)	98.11 ± 13.42	97.51 ± 14.19	98.87 ± 13.58	0.517	0.066	0.078
PP (mmHg)	51.51 ± 13.97	53.24 ± 17.33	54.50 ± 16.43	**0.032**	0.157	0.055

Data are shown as means ± SD. *P* value is derived by analysis of variance (ANOVA).

^a^ *P* values compared DD genotype with II genotype.

^b^ *P* values compared ID genotype with II genotype.

^c^ *P* values compared DD + ID genotypes with II genotype.

BMI: body mass index; WHR: waist-to-hip ratio; FPG: fasting plasma glucose; HbA_1c_: glycated hemoglobin; TC: total cholesterol; TG: triglycerides; LDL-C: low-density lipoprotein cholesterol; HDL-C: high-density lipoprotein cholesterol; SBP: systolic blood pressure; DBP: diastolic blood pressure; MAP: mean arterial pressure; PP: pulse pressure.
